# Assessing the Effect of High Performance Inulin Supplementation via KLF5 mRNA Expression in Adults with Type 2 Diabetes: A Randomized Placebo Controlled Clinical Trail

**DOI:** 10.15171/apb.2018.005

**Published:** 2018-03-18

**Authors:** Abed Ghavami, Neda Roshanravan, Shahriar Alipour, Meisam Barati, Behzad Mansoori, Faezeh Ghalichi, Elyas Nattagh- Eshtivan, Alireza Ostadrahimi

**Affiliations:** ^1^Department of Nutrition, School of Nutrition, Tabriz University of Medical Sciences, Tabriz, Iran.; ^2^Cardiovascular Research Center, Tabriz University of Medical Sciences, Tabriz, Iran.; ^3^Department of Molecular Medicine, Nutrition Research Center, Tabriz University of Medical Sciences, Tabriz, Iran.; ^4^Department of Nutrition, School of Nutrition, Shahid beheshti University of Medical Sciences, Tehran, Iran.; ^5^Immunology Research Center, Tabriz University of Medical Sciences, Tabriz, Iran.; ^6^Nutrition Research Center, Tabriz University of Medical Sciences, Tabriz, Iran.

**Keywords:** Diabetes, Inulin, KLF5, miR-375, Fasting plasma glucose

## Abstract

***Purpose:*** The worldwide prevalence of metabolic disorders such as diabetes is increasing rapidly. Currently, the complications of diabetes are the major health concern. The aim of this study was to investigate the effect of high performance (HP) inulin supplementation on glucose homeostasis via KLF5 mRNA expression in adults with type 2 diabetes.

***Methods:*** In the present clinical trial conducted for a duration of 6 weeks, 46 volunteers diabetic patients referring to diabetes clinic in Tabriz, Iran, were randomly assigned into intervention (n= 23, consuming 10 gr/d HP inulin) and control groups (n= 23, consuming 10 gr/ d starch). We assessed glycemic and anthropometric indices, blood lipids and plasmatic level of miR-375 as well as KLF5 mRNA expression before and after the intervention.

***Results:*** Findings indicated that inulin supplementation significantly decreased fasting plasma glucose (FPG) in comparison to the placebo group (P<0.001). Also Intra-group and between group results showed that inulin supplementation resulted in significant decrease in KLF5 mRNA expression in peripheral blood mononuclear cells (PBMCs) (Fold change: 0.61± 0.11; P-value= 0.001) and significant increase in plasmatic level of miR-375 (Fold change: 3.75± 0.70; P-value=0.004).

***Conclusion:*** Considering the improvements of FPG level in diabetic patients, it seems that HP inulin supplementation may be beneficial in controlling diabetes via the expression of some genes. However, further studies are needed to achieve concise conclusions.

## Introduction

Diabetes mellitus (DM) as a complex metabolic disorder influenced by various environmental and genetic factors has become a common health problem in the entire world.^1^ According to recent reports it is estimated that by the year 2030, at least 366 million people will suffer from diabetes.^2^ Recent scientific advances point to manipulation of gut microbiota as contributing factors for preventing or controlling diabetes.^2,3^

The intestinal microbiota is a vital organ with trillions of commensally microorganisms which is involved in host metabolism. Nowadays, dietary components, particularly prebiotics are considered as functional foods that provide beneficial health effects on the intestinal tract. Prebiotics are defined as “ non-viable food components that confer health benefits on the host in association with modulation of the microbiota”.^4^

Inulin- type fructans are a kind of prebiotic fibers that have received much attention in the last decade. Inulin (a mixture of fructo oligo- and polysaccharides) is a very interesting functional ingredient, present as storage carbohydrate in more than 30,000 vegetables and fruits such as garlic, chicory root, wheat and banana.^5,6^ High performance (HP) inulin, the highly refined kind of inulin, is the average rate of polymerization which consists of 25 monosaccharide units. This form of inulin has many advantages with minimum gastrointestinal side effects such as abdominal tension.^7,8^ The beneficial health effects of inulin-type fructans have been previously studied. It has been showed that inulin may modulate glucose homeostasis by direct and indirect mechanisms. Alteration in gene expression and its effect on the gut microbiota at different taxonomic levels are few of the beneficial effects of inulin.^9,10^

Recent evidences showed an elaborate network of certain transcription factors coordinate with the expression of hundreds of genes which are responsible for the beneficial effects of inulin.^11,12^ The Kruppel Like Factor (KLF) is a kind of zinc finger transcriptional factor which encodes proteins that bind directly to a specific recognition motif in the promoters of target genes. KLF5, a member of the KLF family known as Gut-Enriched Kruppel-Like Factor (GKLF) has been characterized as a transcription factor which is expressed in high amounts in the cells of the intestinal epithelium.^13^ Previous studies reported an intense association between KLF5 overexpression and some metabolic disorders such as cardiovascular disease and diabetes.^14^ It has been proved that the translation of KLF5 is controlled by microRNAs (miRNAs).^15^

MiRNAs are small (~ 22 nucleotides), single strand, noncoding RNAs which are important regulators of gene expression via base pairing with 3´- untranslated regions of messenger RNA (mRNA).^16^ In fact, MiRNAs could possibly lead to degradation of mRNA or protein translation inhibition.^17^ Pioneering studies showed that the expression of miR-375 is able to prohibit the translation of KLF5.^18^ As the miRNA expression is very high in the human intestine,^19^ we supposed that mediation of the gut microbiota with inulin supplementation may promote glucose homeostasis via overexpression of miR-375 in adult diabetic patients. Thus, the objective of this study was to evaluate the effect of HP inulin supplementation on glucose homeostasis via KLF5 mRNA expression in type II diabetic patients in the form of a randomized, double-blind, placebo-controlled clinical trial.

## Material and Methods

### Participants 

In the current randomized, double-blind, placebo-controlled trial, 46 volunteer diabetic adult patients referring to diabetes clinics in East Azerbaijan, Iran, during September 2016 and November 2016, were recruited. With Confidence Interval: 95% & Power: 90%, calculation of the sample size was accomplished based on the fasting insulin parameter.^20^ The formula: n = [(Z1 – α/2 + Z1 – β) 2 (SD12 +S D22)]/Δ2 was used to estimate the 23 samples allocated for each group while considering 6 patients for withdrawal. The inclusion criteria were having diabetes mellitus for more than 6 months; aged 30 to 50 years; body mass index) BMI) greater than 25 and less than 35 kg/m^2^. Exclusion criteria were having kidney disease; liver failure; heart failure; rheumatic diseases inflammatory diseases of the gastrointestinal tract; lactose intolerance; insulin injection and consuming drugs such as: estrogen, progesterone, corticosteroids; smoking; breast feeding and pregnancy; vitamin, mineral, omega-3 and antibiotic supplementation for three weeks before the beginning of the study. These subjects (46 patients) were randomly allocated using randomized block procedure, to one of the 2 treatment groups (A, or B) by computer-generated allocation schedule (Random Allocation Software) in which A was inulin group, B was placebo. Participants were also matched by type of consumed drugs (glucose lowering and anti- hyperlipidemia drugs) and disease duration in this trial.

### Study design

After stratifying patients based on gender and age, subjects were randomly allocated into HP inulin (n=23) and placebo groups (n=23). The randomization process was not disclosed to the researchers and diabetic patients until the main analyses were completed. Also, during the study, none of the researchers and patients was aware of the drug randomization procedure. A study technician accomplished the randomization allocation procedure and allocated the participants into two groups. The HP inulin group received 10 g per day HP inulin powder (Sensus, Borchwef 3, 4704 RG Roosendaal the Netherlands) and the placebo group obtained 10g starch powder as placebo for 6 consecutive weeks. The components used for supplementation were sequenced into equal doses (5 grams) which were prescribed to consume before breakfast and dinner for 6 weeks. HP inulin powder and placebo (starch powder) were manufactured by Sensus Company, Netherlands. The HP inulin powder and placebo were in the same appearance such as colour, shape and packaging, which were coded by the producer to guarantee blinding. The participants were encouraged to avoid changing the dose and drugs consuming in order to prevent potential effects on the results of the study. Compliance to the HP inulin was assessed via asking participants to return the medication packages. Subjects were controlled weekly for possible side effects.

### Physical activity and dietary intake assessments 

For evaluating physical activity level before and after the intervention The International Physical Activity Questionnaire (IPAQ) was used for assessing physical activity level at baseline and end of intervention. According to the categorical scoring guidelines of the concluded form of IPAQ, participants were classified as highly, moderate and/or low physical activity level. we grouped our participants into high, moderate or low physical activity levels.^21^ Dietary intake was determined using a 24-hour food recall method for 2 average working days and 1 weekend day a week before and at the end of intervention. The dietary recalls were analyzed using the Nutritionist IV software (First Databank, San Bruno, CA, USA) adjusted for Iranian foods. Subjects were informed to continue their usual intake and physical activity until the end of the trial.

### RNA extraction and quantitative real-time PCR for gene and miRNA

Peripheral blood mononuclear cells (PBMCs) separation was accomplished by Ficoll-Histopaque solution gradient (ficoll- paque, GmbH) centrifugation. Total RNA was extracted from PBMCs using ambion Trizol LS reagent. Thermo Fisher scientific revertaid first strand cDNA synthesis kit was used for the synthesis of cDNA. The level of KLF5 mRNA were evaluated by SYBR Green Master mix (Thermo Fisher Scientific, USA). The primer sequences were designed using PrimerBank. The amount of the mRNA normalized against the β-actin mRNA and -2^ΔΔC^_T_ method^22^ was used for relative mRNA abundance. For miR-375 expression evaluation, complementary DNA was synthesized from isolated total RNA obtained from 200-µL plasma using the Universal cDNA synthesis kit (EXIQON, Denmark). Quantitative reverse transcription PCR (qRT-PCR ) was prepared using ExiLENT SYBER Green Master mix (Exiqon) against LNA based primer sets (Exiqon) and comparative -2^ΔΔC^_T_ method was used to prove the relative quantitative level of miRNAs using Endogenous Control Primer miR-191 (Exiqon) for miRNA normalization.^23,24^ All samples were run in duplicate. Fold change of the parameters was computed as relative expression post intervention/control. The primers sequences of KLF5, β-actin, miR- 375 and miR-191 are illustrated as following: KLF5: Forward TCATCTTTCTGTCCCTACCC, Reverse TCCATTGCTGCTGTCTGA, Forward GGTGAAGGTGACAGCAGT, Reverse TGGGGTGGCTTTTAGGAT* Hsa*-*miR-375* (5'-3') UUUGUUCGUUCGGCUCGCGUGA, *Hsa*-*miR-191*-5p (5'-3') CAACGGAAUCCCAAAAGCAGCUG.

### Anthropometric measurements

At the onset and end of the trial, anthropometric indices such as body weight (BW), height, waist and hip circumferences (WC and HC respectively), waist to hip ratio (WHR) and body mass index (BMI) were recorded. Weight was measured via a calibrated scale (Itin Scale Co., Inc. Germany) with least clothing and 0.1 kg accuracy. Also, height was measured in a standing position next to ruler attached to the wall. For calculating BMI, the following equation was used: weight (kg) / height^2^ (m). Waist circumference (WC) was obtained by measuring the smallest area below the rib cage and above the umbilicus. Standing HC was measured at the inter trochantric level.^25^ Waist to hip ratio (WHR) was measured by dividing mean WC to mean HC.

### Biochemical Assessment

For biochemical assessment, 7 ml blood sample was collected at baseline and at the end of the trial after 12h overnight fasting for measuring fasting plasma glucose (FPG), fasting insulin, glycated hemoglobin (HbA1c), total Cholestrol (TC), Triglycerides (TG), High density lipoprotein cholesterol (HDL-C) and Low density lipoprotein cholesterol (LDL-C). Auto-analyzer (Mindray Auto Hematology Analyzer, China) was used for biochemical analysis as well as platinum enzyme-linked immunosorbent assay (ELISA) kit (Monobind, Iran) for measuring fasting insulin. Also, NycoCard kit (NycoCard, Norway) was used for measuring HbA1c. Based on the Homeostatic model assessment of insulin resistance (HOMA-IR method, insulin resistance was measured: Fasting Glucose (mg/dL) × fasting insulin (mU/L)/450.

### Statistical analysis

The Kolmogorov-Smirnov test was used for testing the normal distribution all of variables. Numerical variables were compared by Student t test and reported as mean ± standard deviation (SD). The Pearson chi-square test was used for comparing categorical variables and reported as number (%). Within group comparisons were determined by paired-sample t test. After adjusting for baseline values, Analysis of Covariance (ANCOVA) was applied to identify any differences between two groups at the end of the study. Statistically significant variables had p-value less than, 0.05 and were analyzed using the SPSS software version 23 (SPSS Inc., Chicago, Illinois, USA).

## Results

**Figure 1:**
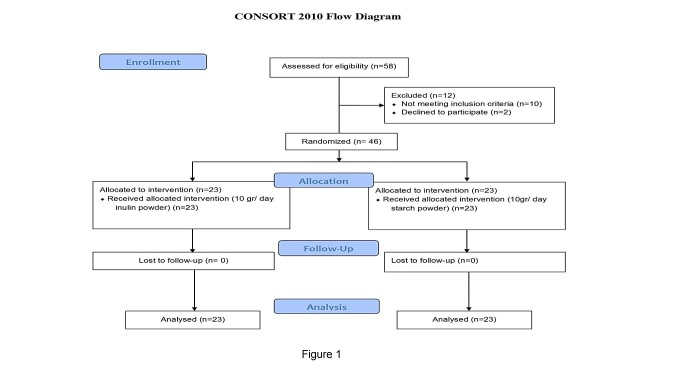
Trial flow diagram

The study flowchart is shown in Figure 1. A total of 46 diabetic patients completed the study and were interred in the final analyses. No adverse side-effects were reported by diabetic patients following the HP inulin or placebo supplementation. General characteristics (demographic variables and baseline values of anthropometric indices) of the participants are presented in Table 1. None of the demographic and anthropometric variables were different between the study groups, at baseline (P>0.05). As specified in Table 2, no differences were seen in the percent changes of few of the variables such as HbA1c, Weight, BMI, anthropometric indices (WC, HC and WHR) and blood lipids (TC, TG, HDL-C, LDL-C) after Inulin supplementation compared to the placebo group (P>0.05). However, Inulin supplementation significantly decreased FPG in comparison to the placebo group (P<0.001). Also, a statistically insignificant increase was observed in fasting insulin level after inulin supplementation. Intra-group statistical analysis indicated that Inulin supplementation significantly reduced FPG, WC and HC (P<0.05). Inulin supplementation significantly decreased WC from 97.47± 8.41 to 96.28±8.03 (p=0.009).

Dietary intake of study participants is shown in Table 3‏ demonstrates subject’s dietary intake. Within group ad between group differences for dietary intake of energy and macro-nutrients such as carbohydrates, proteins, fats and dietary fiber was not statistically significant. There were no within-or between-group differences observed for dietary intake of total energy, carbohydrates, proteins, fats and dietary fiber.

**Figure 2:**
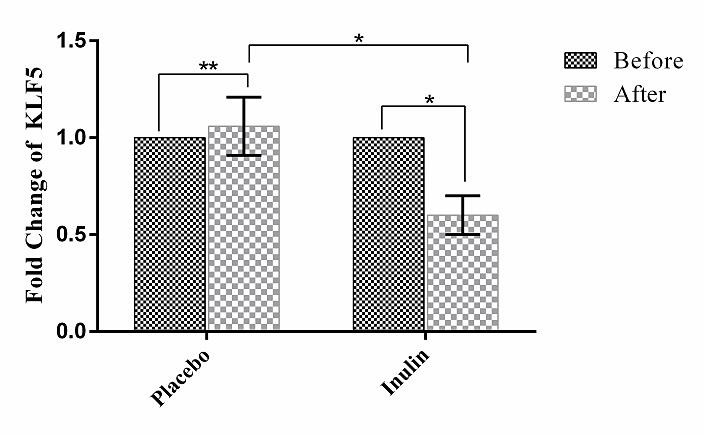
The PBMC levels of KLF5. X-axis represents the study groups and Y-axis shows fold changes of KLF5. Statistical analysis was done by One Sample T test and Independent Sample T test. Each point representsmean±SD. * P-value< 0.05; ** P-value> 0.05.

**Figure 3:**
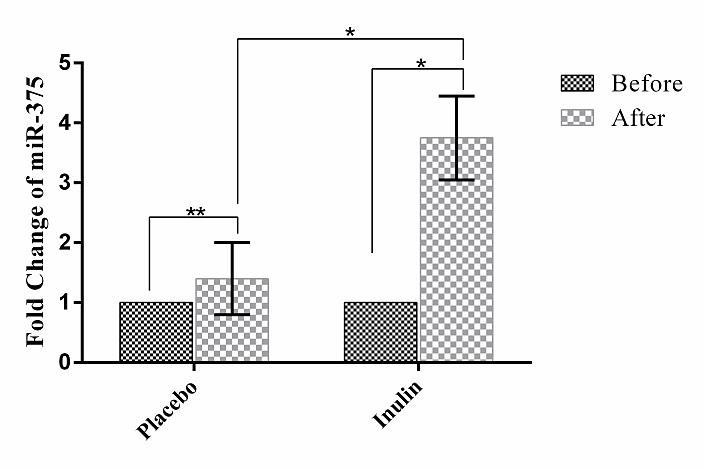
The plasma levels of miR-375. X-axis represents the study groups and Y-axis shows fold changes of miR-375. Statistical analysis was done by One Sample T test and Independent Sample T test. Each point represents mean±SD. * P-value< 0.05; ** P-value> 0.05.

Intra-group and between-group statistical analysis revealed that Inulin supplementation resulted in significant decrease in KLF5 mRNA expression of KLF5 (Fold change: 0.61± 0.11; P-value= 0.001) (Figure 2). Additionally, Inulin supplementation significantly increased miR-375 level (Fold change: 3.75± 0.70; P-value=0.004) (Figure 3).

**Table 1:** Baseline characteristics of the study subjects

**Variable**	**HP inulin group** **(n=23)**	**Placebo group** **(n=23)**	**P-value** ^b^
Male/Female	10/13	10/13	1.00
Age(year)	41.50±6.27	42.73±5.95	0.509
Weight(kg)	81.87± 11.46	79.91± 14.60	0.624
Height(cm)	168.68± 8.83	164.09± 10.10	0.116
BMI(kg/m^2^)	27.71± 4.60	28.79±4.77	0.444
WC(cm)	97.47± 8.41	93.84± 11.16	0.467
HC(cm)	108.04± 7.39	104.50± 10.70	0.455
WHR	0.90±0.08	0.88±0.07	0.455
Diabetes duration(year)	8.78± 4.67	9.86± 4.95	0.546
Physical activity level n(%)			0.459
Low	14(60.87)	15(65.23)	
Moderate	7(30.44)	7(30.43)	
High	2(8.69)	1(4.34)	

Abbreviations: BMI, body mass index; WC, waist circumference; HC, hip circumference; WHR, waist To hip ratio. ^a^Variables are expressed as mean ± SD.‏ And number (percentage). ^b^*p*-values resulted from independent t tests for quantitative and Chi-square for qualitative variables between the two groups.

**Table 2:** The effect of HP inulin supplementation on anthropometric indices and biochemical in patient with diabetes.

**Variable**	**HP inulin group(n=23)**		**placebo group(n=23)**		**P-value** ^a^
	**mean±sd**	**Change**	**mean±sd**	**Change**	
**Weight (kg)**		-0.61		-0.47	0.317
Baseline	81.87± 11.46		79.91± 14.60		
End of trial	81.46± 11.39		79.40± 13.91		
P-value^b^	0.362		0.259		
**BMI(kg/m** ^ 2 ^ **)**		-0.710		-0.62	0.792
Baseline	30.37±2.47		30.86±2.41		
End of trial	30.15±2.73		30.64±2.24		
P-value^b^	0.104		0.084		
**WC(cm)**		-1.17		-0.003	0.952
Baseline	97.47±8.41		93.84±11.16		
End of trial	96.28±8.03		93.68±9.98		
P-value^b^	0.009		0.846		
**HC(cm)**		-1.93		-1.17	0.687
Baseline	108.04±7.39		104.50±10.70		
End of trial	106.01±8.23		103.22±10.47		
P-value^b^	0.004		0.061		
**WHR(cm)**		-0.21		-1.28	0.192
Baseline	0.90±0.08		0.88±0.07		
End of trial	0.90±0.08		0.87±0.05		
P-value^b^	0.744		0.135		
**Insulin(µU/ml)**		7.54		-0.57	0.817
Baseline	4.99±1.41		5.42±1.56		
End of trial	5.20±1.75		5.38±1.56		
P-value^b^	0.546		0.598		
**FPG(mg/dl)**		-7.46		1.93	<0.001
Baseline	130±36.25		127.64±24.19		
End of trial	119.28±30.75		130.36±27.85		
P-value^b^	0.001		0.216		
**HOMA-IR**		1.05		1.47	0.815
Baseline	1.53±0.53		1.71± 0.60		
End of trial	1.55±0.69		1.72± 0.61		
P-value^b^	0.974		0.568		
**HbA1c (%)**		-0.69		-2.52	0.128
Baseline	8.04±2.45		7.02±1.60		
End of trial	7.62±1.85		7.79± 1.29		
P-value^b^	0.389		0.156		
**TG(mg/dl)**		-0.80		-0.40	0.136
Baseline	177.16±72.02		186.73± 74.64		
End of trial	168.58±53.50		185.45± 7.74			
P-value^b^	0.299		0.402		
**TC(mg/dl)**		-7.77		2.97	0.819
Baseline	201.38±73.43		174.27± 42.35		
End of trial	178.24±55.01		176.18±31.02			
P-value^b^	0.122		0.742		
**HDL-C(mg/dl)**		4.79		1.49	0.388
Baseline	41.18±9.23		41.64±8.75		
End of trial	42.95±9.19		42.09± 9.42		
P-value^b^	0.091		0.681		
**LDL-C(mg/dl)**		-8.32		6.52	0.070
Baseline	111.81±48.54		107.20±30.96		
End of trial	97.57±44.05		112.32±32.60		
P-value^b^	0.183		0.169		

Abbreviations: ANCOVA, analysis of co-variance; BMI, body mass index; HDL-C, High density lipoprotein cholesterol; LDL, low density lipoprotein cholesterol; TG, triglycerides; TC, total cholesterol; WC, waist circumference; HC, hip circumference; WHR, waist to hip ratio; FPG, fasting plasma glucose; HbA1c, glycosylated hemoglobin; HOMA-IR, homeostasis model assessment of insulin resistance;^a^ obtained from ANCOVA adjusted for baseline value. ^b^obtained from paired T test.

**Table 3:** Dietary intake of study participants at Baseline and End of trial^a^

**Variable**	**HP inulin group (n=23)**	**Placebo group(n=23)**	**P-value** ^b^
**Energy(Kcal/day)**			
Baseline	1680.24±387.81	1739.57±421.05	0.603
End of trial	1786.84±495.04	1666.15±458.37	0.370
P-value^c^	0.213	0.505	
**Carbohydrate(g/day)**			
Baseline	242.92±62.84	255.25±77.19	0.536
End of trial	259.25±80.20	242.38±60.06	0.398
P-value^c^	0.18	0.58	
**Protein(g/day)**			
Baseline	66.95±19.01	70.73±19.83	0.491
End of trial	74.35±24.41	67.69±19.14	0.283
P-value^c^	0.13	0.45	
**Fat(g/day)**			
Baseline	50.73±15.90	51.05±16.50	0.944
End of trial	50.55±13.38	47.56± 18.79	0.518
P-value^c^	0.96	0.37	
**Dietary fiber(g/day)**			
Baseline	18.35± 6.62	17.60± 4.60	0.519
End of trial	17.92± 3.95	16.95±4.30	0.109
P-value^c^	0.231	0.401	

^a^Variables are expressed as mean ± SD,). ^b^*p*-values resulted from independent T tests.^c^obtained from paired T test.

## Discussion

Our findings provide strong evidence for modulation of glycemic indices via miR-375 as an important regulator for KLF5 mRNA expression in type 2 diabetic patients after HP inulin supplementation. The findings of the present study indicated that HP inulin supplementation decreased FPG and KLF5 mRNA expression significantly. Interestingly, miR-375 expression increased remarkably in the intervention group compared with the placebo group. Additionally, our study revealed that the levels of FPG, WC and HC decreased in inulin group compared to the placebo one.

Based on our understanding, the present study is the first study that investigated the effects of HP inulin supplementation on KLF5 mRNA expression via miR-375 up-regulation. The decrease of FPG level in our study is in agreement with earlier results.^26,27^ In contrast, in some previous studies no beneficial effects were reported for FPG level after inulin- type fructans supplementation in diabetic patients.^28,29^ These conflicting effects could be due to different ethnic background, various doses and time intervals of inulin supplementation. Regarding the effect of inulin supplementation on fasting insulin, HbA1c and HOMA-IR indices we did not observe any significant changes in line with pioneering studies.^30,31^ According to a new systematic review and meta- analysis, there were no significant improvement in fasting insulin after inulin-type fructans’ supplementation.^32^

Similar to few previous studies^8,33,34^ we observed no statistical significant changes in lipid profile following HP inulin supplementation. Nevertheless, in few studies positive effects of prebiotics on lipid profile were reported.^35,36^ The contradictory results obtained from several studies may be due to the diversity in type and dose of supplements and differences in baseline values of lipid profile.

Other studies have shown that fructooligosaccharides’ consumption was associated with anthropometric indices improvement and the promotion of weight loss.^20,37-40^ However, conflicting results have also been reported.^30^ In this study inulin supplementation resulting in WC, HC decreases in intra-group analysis but in between-group assessments no significant differences were observed.

An interesting finding was the effect of inulin supplementation on KLF5 mRNA expression. It has been previously indicated that plasmatic level of miR-375 is considered as a biomarker for β-cell function. This miRNA is a key regulator for securing intestinal epithelium safety and multiple metabolic processes via controlling various gene expression.^41^ Indeed, KLF5 is a target gene for miR-375 and has been implicated in glucose homeostasis.^42^ In the present study, we focused on conserved intestinal evolutionary miRNA (miR-375) in order to evaluate glucose homeostasis. In fact, we found a relation between modulation of gut microbiota and overexpression of miR-375 following inulin consumption by suppressing the KLF5 expression in PBMCs. Interestingly, KLF5 is one of the essential transcriptional factors communicating with inﬂammatory conditions and may contribute to the improvement of glucose homeostasis and diminution of inflammatory cytokines like Tumor necrosis factor alpha (TNF-α) through chemoattractant protein-1 (MCP-1) and transcription factor Nuclear factor-kappa B (NF- κB) pathways.^43^

Although the exact mechanisms by which inulin- type fructans act on glucose metabolism remain unclear, however several proposed mechanisms have been reported, including:

Ι. Changes in gut microbiota composition particularly increased the number of bifidobacteria and butyrate- producing colon bacteria after feeding inulin.^44^

ΙΙ. Fermentation of inulin- type fructans in the large bowel into short chain fatty acids(SCFAs) such as acetate, butyrate and propionate modulate inﬂammation by preventing the production of TNF-α and NF- κB.^45^

ΙΙΙ. Butyrate and propionate induce intestinal gluconeogenesis which thereby improves glucose homeostasis.^32^

IV. Increased production of short chain fatty acids in colon may control the expression of some genes and miRNAs which are involved in glucose homeostasis.^46^

No study is without limitations and the limitations of this study include the small sample size and lack of measurement of some related factors such as inflammatory markers, serum levels of short-chain fatty acids and other genes expression related to glucose homeostasis. The strength of this study is that it was the first study investigating the beneficial effects of inulin supplementation on KLF5 and miR-375 expression in diabetic patients. In order to obtain further conclusive results and determine the exact related mechanisms, more studies with larger sample sizes are needed.

## Conclusion

According to the effectiveness of inulin supplementation on glucose homeostasis through effective mechanisms like genes expression, this survey can be considered as a novel therapeutic approach in controlling diabetes. Our results revealed new insights into how KLF5 functions play a role in controlling diabetes. The newly identified KLF5- miR-375 pathway may contribute to diabetes progression. Inhibiting KLF5 may be a potential pharmacological intervention in controlling diabetes. With descriptions above, inulin supplementation maybe considered an adjunctive therapy for diabetic patients.

## Acknowledgments

The authors would like to thank all the study participants, as well as nurses and doctors in the diabetes clinics in East Azerbaijan, Iran for their collaboration. The present study did not achieve any grants.

## Ethical Issues

The Ethics committee of Tabriz University of Medical Science (Ethic code: IR.TBZMED.REC.1395.671) approved the research protocol of the study and a written informed consent document was obtained from all patients. The study was registered in the Iranian Registry of Clinical Trials website (IRCT ID: IRCT201610212017N31).

## Conflict of Interest

The authors would like to gratitude the individuals who participated in this study.

## Abbreviations

HP: high performance; KLF5: kruppel like factor 5; FPG: fasting plasma glucose; PBMCs: Peripheral blood mononuclear cells.
